# Collectanea

**Published:** 1829-12

**Authors:** 


### COLLECTANEA.

Floriferis ut apes in saltibus omnia libant,
Omnia nos, itidem, depascimur aurea dicta.

### PHYSIOLOGY.

*On the Action of the Spinal Marrow in Respiration.* By M. Flourens.

Every body knows the opinion of the celebrated Le Gallois, who was led by a series of experiments, then entirely new, to place the seat of the principle of the motions of the heart in the spinal marrow.

M. Flourens shewed, in 1823, first, that the circulation, which, in adult animals, is instantly stopped by the destruction of the spinal marrow, on the other hand, survives its destruction a certain time in new-born animals; secondly, that, even in adult animals, (and this had already been determined by Dr. Wilson Philip,) the circulation survives the destruction of the spinal marrow, provided the respiration be kept up by insufflation. Thus, in the young animal, in which respiration is less necessary to the circulation, the spinal marrow is also less necessary. It is therefore especially because it is subservient to respiration, that the spinal marrow is subservient to circulation.

Whence it follows that, if there were an animal in which the respiration might be completely disconnected, at least for a certain time, from the spinal marrow, the circulation might also be completely disconnected from it.

This animal is the fish. "I have shown," said M. Flourens, "by previous experiments, that the spinal marrow may be entirely destroyed in fishes, without destroying the respiration; seeing that it is no longer from the spinal marrow, as in the other classes, but from the medulla oblongata alone, that in these animals the nerves of the respiratory mechanism take their origin."

The spinal marrow may equally be destroyed in fishes without destroying the circulation.

"I successively destroyed, in several carps and barbels, the whole spinal marrow, without touching the medulla oblongata. In all these fishes, the respiration and circulation continued for a long time; the motions of the trunk and appendages alone disappeared, but the head and the region of the opercula continued to move as usual; and the circulation still went on, even at the extremity of the trunk, more than half an hour after the total destruction of the spinal marrow."

On the other hand, the author always found in the other classes the circulation survive the destruction of all the parts of the spinal marrow which the respiration survived: the destruction in birds, for example, of the lumbar portion, and that of the lumbar and costal portions in quadrupeds.

Thus, therefore, 1. There may be destroyed, without detriment to the circulation, all the parts of the spinal marrow, which may be destroyed without detriment to respiration; and, when the spinal marrow may be entirely destroyed without injuring the latter, as in fishes, it may be entirely destroyed without injuring the former.

2. The spinal marrow has therefore but a relative and variable action upon the circulation as upon the respiration.

3. It is therefore especially because it exerts an influence upon the respiration, that the spinal marrow influences the circulation; and it is by the same parts that it acts upon each.

4. It is not in it, therefore, that the sole principle of the circulation exists.

Where, then, does this principle reside? The author intends, in a future memoir, to point out the parts in which his experiments have led him to place it, and to show the mode according to which it is distributed in them.—*Edinburgh New Philosophical Journal.*

---

*Notice respecting a Pigeon which continued to live two days without Brain or the upper part of the Spinal Marrow.*—M. Desportes, a physician, lately sent to the Academy of Sciences of Paris an account of an observation in which he saw a young pigeon live for two days in its shell, of which it could not rid itself; as well as some time after, although the brain and upper part of the spinal marrow were wanting. The author of the letter, deceived by the accounts given in some journals, had imagined this observation to be in contradiction to what M. Flourens had announced with respect to the influence of the spinal marrow upon respiration. M. Flourens remarked, that the important fact observed by the author is in no degree opposed to the inferences deducible from his experiments. A report is to be made to the Academy respecting M. Desportes's observations.—*Ibid.*

---

### PATHOLOGY.

*Rupture of the Hepatic Duct.* By Dr. Wolff.—A woman, sixty years of age, of a good constitution, the mother of seven children, had many years ago been attacked with jaundice; since which time she had been subject to colic, nausea, and vomiting. These attacks, however, were neither long nor severe. They required nothing more than simple remedies.

July 1st, Dr. W. was called to her in haste. She was suffering from violent spasm of the stomach, and crying out loudly. In a few minutes the spasm ceased, and the patient said that for some days she had been troubled with her accustomed colicky pains, but at first in a slight degree. They gradually became very violent; she threw up the food she had taken at dinner, but was not relieved, and the remedies she had before found beneficial were now had recourse to in vain. She had several returns of the pain, in a violent degree, while Dr. W. remained with her. Her pulse was nearly natural; heat of skin not increased; tongue clean; bowels open, and appearance of stools healthy; urine as limpid as water. During the intervals of pain, there was not the slightest tenderness of the abdomen on pressure. Fifteen drops of tincture of opium relieved her. In the night she had several severe attacks, and was again relieved by an opiate. In the morning she was awoke from her sleep with dreadful pain. Her belly was now swoln, tense, and very sensible; hands and feet icy cold; face covered with cold sweat; pulse rapid and small. She complained of great thirst, and every thing she took was immediately thrown from the stomach. Some internal rupture was feared, and a very doubtful prognosis was given. The case of Admiral Wassenaer, which was seen by Boerhaave, and is reported by Zimmerman, occurred to the mind of Dr. W.* Thirty leeches were applied to the lower part of the belly. The pain, however, increased, and every moment the anxiety and restlessness of the patient became greater. Her intellect was clear. She had constant but ineffectual desire to pass a motion. As the leeches had produced no benefit, blisters were applied, and opium was given, but without avail. A linseed injection procured a copious feculent stool, and she passed a small quantity of turbid urine. In the course of a few hours the pulse could not be felt. The coldness of the extremities had increased; the thirst and vomiting continued; the swelling of the abdomen was greater; the breathing was very quick. She retained her faculties to the last, and her muscular force was surprising. About four o'clock in the morning, twenty-four hours after the commencement of the violent spasms, she died.

*Dissection.* In the cavity of the abdomen was found about three pints of blood mixed with bile. The intestines were inflamed in different parts. Upon the omentum minus there was a spot of a dark colour, several inches in diameter; and, upon prosecuting the examination carefully, the hepatic duct was found ruptured transversely. The torn ends of the canal floated in a mass of coagulated blood, in which could be easily distinguished a recent coagulum, and one of previous formation, which was now much altered in appearance. Liver healthy. Gall-bladder contained a natural quantity of bile, and eight pointed calculi, each about the size of a pea, of a brownish green colour. The smallest of these calculi was in the cystic duct, which it did not however completely close, nor altogether prevent the exit of bile.

It is very probable that the hepatic duct had been inflamed when the patient was attacked with jaundice, many years ago, and that the extravasation had then taken place. The biliary calculi also were possibly formed at the same time. The violent attack on the 1st of July appeared purely spasmodic, and apparently arose from the irritation produced by the calculus in the cystic duct.—*Journal Complementaire*, Sept. 1829.

---

*Polypus attached to the Fundus Uteri.*—M. Lisfranc lately found, upon dissecting a woman who had died of peritonitis, a large fleshy polypus attached by a broad pedicle to the fundus uteri. The fact is curious, inasmuch as it shows, contrary to the opinion of some authors, that polypi may arise from that part of the womb.—*Ibid.*

* Admiral Wassenaer died from laceration of the œsophagus. The symptoms had been so obscure during his illness, that Boerhaave, and another physician of great ability, had not the slightest notion of the nature of the malady. The case of Admiral A. is detailed at great length by Zimmerman, in his Treatise on Experience in Physic, vol. i. p. 233, English transl.—Editors.

*Case of the Bite of a Tarantula.* By GASTANO SPIZZIRI, D.M. (*Osservatore Medico*, No. 19.)

Towards the end of July, 1826, a man, named Antoniello, at Marano, twenty years of age, of a tranquil temperament, saw two insects, about the size of large spiders, and resembling them in shape, upon one of his boots: he paid no attention to them, but presently felt a sharp burning pain in the left arm, near the radial artery. There was no appearance of a bite; he only observed a drop of yellow humour, which he immediately wiped off. The painful sensation, notwithstanding, spread with the celerity of lightning to the armpit, and downwards towards the knee, and was also felt in the same direction on the opposite side. It quickly spread to the bones, which felt as if torn by pincers. Not being able to stand, he laid on the ground. His whole frame was convulsed; his body became cold, and the surface was covered with a yellow sweat; nostrils much dilated. In this condition his companions conveyed him to Marino. It should be remarked that, during the journey, the convulsive pain was suspended; but, when the mule stopped, the patient felt inclined to dance. A surgeon directly burnt the part with a hot iron, which produced no relief: the burn was not even felt.

The patient's father having great confidence in the empirics of Mendicino, known by the name of Ciraulari, sent for the cleverist of them, who, without loss of time, employed his superstitious charms. He put his right hand first upon the left thigh, and then upon the other; and the convulsion ceased first on the left side, and then on the right, as if the hand of Medusa had been applied.

It is left to the reader to decide whether the result is to be attributed to the effect of moral influence or not. It is certain that the patient of the village Esculapius found himself cured on the third day, having previously taken a bath of wine, in which had been boiled, in a copper kettle, his heroic herbs, amongst which was rosemary. The only internal remedy had been a glass of pennyroyal water, sweetened with sugar, which made his stomach uneasy.

Vincent Vena, of Marano, forty years of age, of rather an irritable temperament, was bitten in the same part, at the same place, by a little insect of a black colour, which, though killed with the other hand, was discovered to be a tarantula. He immediately felt, as in the preceding case, a sharp pain, which spread from the bitten part to the corresponding armpit, describing a single line, which became a yellow stripe; but he felt nothing in the other parts of his body, only a tormenting inclination to change of place and to dance. He submitted himself to the same empiric, who, by the same means, cured him at the end of three days.

M. S. Spizziri adds, in a note, that there is reason to believe that the plant with which the village physician of Mendicino performed these and other well-authenticated prodigies, is the Branck-ursine, the Aconthus Mollis of LINNÆUS.

---

*Account of the Effects of the accidental Inhalation of the Gas of the Burning Coal in the Wanlockhead Mines.* By WILLIAM WATSON, Esq. Surgeon, Wanlockhead.

The Wanlockhead Mining Company finding it expedient, a few years ago, to place a steam engine on one of their mines for the purpose of lifting the water, had a large turnhead or excavation cut in the rock for the accommodation of the machinery; and, that they might the more easily remove the smoke and other impurities arising from the combustion of the coal, they had a flue constructed of fire-brick, the entire length of which was 456 feet, and the perpendicular division of it 384. This they carried through an old mine, the lower part of which, being rather soft, required to be supported with wood. The flue till lately answered the purpose extremely well; but on Saturday, the 28th March last, the wood supporting the lower part of the excavation in which the flue is placed was observed to be on fire. As fears were entertained that the fire might communicate with the wood in the excavation connected with the machinery, the utmost exertions were made to arrest its alarming progress. These, in so far as the part surrounding the flue was concerned, proved entirely abortive, but were completely successful in preventing the destructive element from reaching the machinery.

The whole of Saturday was spent in fruitless attempts to extinguish the fire, which continued its ravages with unabated fury, and to ascend towards the surface till about twelve o'clock A.M., when the superintendent for the time resolved on closing the top of the flue to prevent the access of atmospheric air, and by that means to cut short the progress of the combustion. The measure in part succeeded, but, about eight o'clock A.M. on Sunday, considerable quantities of smoke were still seen to issue through the dense covering on the top of the chimney; and, as there was reason to apprehend that the mine might be laid under water, the overseers were induced to order thirty of the operatives into the mine to remove their tools. At that period, eleven o'clock, ten hours had nearly elapsed from the time that the air-passages had been closed till the miners again entered the mine, during which period combustion had still gone on to a certain extent: consequently, the greater part of the oxygen in the mine was consumed, and a corresponding quantity of carbonic acid gas, perhaps a little carbonic oxide, was generated, and rendered the surrounding medium highly noxious, and incapable of supporting either combustion or animal life. It was into this immense mass of irrespirable air, then, that thirty human beings precipitated themselves, some to the depth of sixty, others to the depth of 120 feet. Alike ignorant both of the existence and of the noxious quality of the gas, they perambulated the different ramifications of the mine in search of their implements, with the same unconcern as when it had its wonted supply of vital air. The effects, however, soon became evident on all who had thus heedlessly exposed themselves, and occasioned something like a simultaneous movement from every part of the work towards the pit. But this exertion, from accelerating respiration, only increased the evil. Debility made rapid strides, and a number of the poor fellows, after making every possible effort to save their companions, sunk into a state of insensibility themselves. At this period one or two of those who had suffered least with considerable difficulty reached the surface, where a number of their companions were assembled in anxious expectation of the issue. Twenty of these, with laudable intrepidity, hurried into the mine, and had the satisfaction to find that a few had by that time reached a station of comparative safety, though they were extremely weak; but the greater part were still exposed to the deadening influence of the gas. Two were still at the depth of sixty feet, one of whom was lashed to the rope by his companion, who, after performing what he supposed the last friendly office on earth, attempted to leave the death-like scene, but had only reached the distance of eighty feet when he also fell senseless upon the floor of the mine, and was there found by those who went to his assistance. All, by the blessing of Providence, were ultimately rescued from their perilous situation.

The effects produced upon the different individuals were rather curious. Headach, giddiness, tingling of the ears, vomiting, tremor, with extreme debility, were more or less the portion of all. Two were in a state of insensibility for at least forty minutes; six for a shorter period. Three or four were highly excited, and, although it was Sunday, whistled, sung, and talked with the greatest volubility, occasionally threatening to inflict summary punishment on those near them for alleged injuries, not even recognising their nearest relations. In many respects they were very like persons in a state of intoxication from ardent spirits. The circulating system was considerably affected. In two cases the pulse was rather full; but in almost all the others it was soft and yielding; and in the two who were longest exposed, quick (ninety-four or one hundred), small, and very compressible. One man had an attack of convulsions six hours after he had ceased to inhale the gas; a second had diarrhœa, with considerable tenesmus; a third dysuria; a fourth lost all use of his arms from the elbow downwards for several hours; a fifth had colic; several experienced a slight difficulty in breathing, with oppression about the chest. The shortest period of exposure would amount to forty minutes; the longest to one hour and a quarter.

The remedial measures were free exposure to the external air, with a liberal use of diffusive stimuli, the principal of which was ardent spirits. The limbs were occasionally immersed in warm water, with the happiest effects. When nausea was present, a few grains of ipecacuan were administered to excite vomiting; and when torpidity of the bowels was induced, colocynth pills, with castor oil, were given freely.

The individual who had an attack of diarrhœa uniformly experiences the same effects after indulging in ardent spirits.—*Edinburgh Medical and Surgical Journal.*

---

*Case of Hemorrhage after Extraction of a Tooth, from a hereditary Hemorrhagic Tendency.*—A Jew, twenty years of age, thin, cachectic, and pale-featured, was attacked with obstinate hemorrhage after the extraction of the first grinding tooth of the right side of the lower jaw. When Dr. Steinmetz saw him next day, the blood flowed in a continuous stream, and appeared to issue from the lacerated gum, not from the cavity from which the tooth-fangs had been extracted. An attempt was made to arrest the hemorrhage by squeezing into the cavity a mass of charpie sprinkled with styptic powder, and directed the patient to compress it by keeping the jaw closed; but in two hours he complained that the blood began to trickle into his throat. The charpie was therefore renewed, being previously dipped in dilute sulphuric acid. This plan likewise failed; in a short time the blood began again to fill the mouth. It was again tried, however, but without success: two hours after the second pledget was introduced, the blood began to issue from the man's mouth and nose. The charpie was then soaked with concentrated sulphuric acid, and also sprinkled with styptic powder; and, in order to effect exact compression, Lampe's tourniquet for hemorrhage from the tongue was applied to the jaw. During three days this dressing was changed every four or six hours, yet in the end without any advantage. The patient was then ordered likewise to take every other hour from forty to eighty drops of the acid elixir of Haller; and, when this remedy had been persevered in for six-and-thirty hours, the hemorrhage ceased materially. For four days more, however, it returned every evening to such an extent as to induce the patient to resume the use of the acid pledgets.

The hemorrhagic tendency in this instance was derived from the grandfather. Several times, in consequence of a prick with a pin, his grandfather was brought almost to the point of death by hemorrhage. At last he was attacked with pulmonary inflammation, after exposure to cold and fatigue. During this attack he had bloody sputa, though not to any great extent; but on the sixth or seventh day he was attacked with profuse epistaxis, which nothing could check, and which consequently proved fatal. The father had in his youth been likewise several times reduced to a state of extreme exhaustion through hemorrhage produced by trifling injuries, and would have perished but for prompt medical assistance. At the age of sixty-five he had been free from the disease for ten years. The son, Dr. Steinmetz's patient, had been several times before seized with obstinate hemorrhage from trifling causes. Three sisters were entirely free from the hereditary peculiarity, but they imparted it to their children. One of the children was, at the request of the mother, circumcised in presence of the relator, who had extreme difficulty in checking the flow of blood.

These facts confirm the statements of former authors, that the hemorrhagic diathesis descends only to the male branches of a family; that it may be communicated to them through females, without these females having it themselves; and that it ceases in old age.—*Rust's Magazine.*

---

### SURGERY.

*Malignant Pustule cured by Compression.*—M. Godard, of Pontoise, recently communicated this case to the Académie Royale de Médicine. The advantages of the practice he recommends are confirmed by the experience of many esteemed surgeons on the continent, not only in malignant pustule, but in phlegmonous erysipelas, wounds from dissection, and other diseases attended with extensive swelling and inflammation of a limb. In M. Godard's case, a patient was three times attacked with a malignant pustule on the hand. The swelling of the arm, and the other accompanying symptoms, each time yielded to compression of the swollen limb. Cauterization had been previously employed with no avail.—*Journal Complementaire.*

---

*A Case of Aneurism by Anastomosis of the Forehead, treated by the Application of Ligatures.* By B. C. Brodie, F.R.S. Surgeon to the King; and Surgeon to St. George's Hospital. (*Med. Chir. Trans.* vol. xv. part i.)

The disease which Mr. John Bell has described under the name of aneurism by anastomosis* is, according to my experience, of comparatively rare occurrence. Three cases of the kind, however, have been already recorded in

* I have employed the term Aneurism by Anastomosis, because it has become (in this country at least) sanctioned by custom, and for the purpose of avoiding the inconvenience which attends the frequent change of surgical nomenclature. At the same time I must acknowledge that it appears to me to be liable to more than one objection. 1st. I am not aware that it has the Transactions of this Society, and to these I am now induced to add a fourth, the history of which will probably be deemed not devoid of interest, inasmuch as the disease had existed for many years, gradually increasing until it had reached an alarming extent, and, after other modes of treatment had been employed to no purpose, was ultimately cured by a very simple operation, founded on the same principle with that which Mr. WHITE and Mr. LAWRENCE have recommended in cases of the vascular nævus of infants.

Miss ——, in the year 1809, being then about five years of age, received a severe blow on the forehead, in consequence of her having run against the corner of a bedpost. Soon afterwards a small pulsating tumor, not larger than a pea, was observed at the part on which the blow was inflicted. For many years the tumor remained nearly stationary, and, as it produced no inconvenience, it excited but little attention. In the year 1821 it had manifestly increased in size, in consequence of which a surgeon in London was consulted, who attempted to cure the disease by pressure. For this purpose compresses were applied over the tumor, secured by a tight bandage round the head. Under this treatment the patient suffered from a constant and severe pain; and so far was it from being of any service, that, as soon as the pressure was left off, the tumor seemed to grow more rapidly, and the pulsation in it became stronger than before. From this time also there were frequent attacks of intense headach, which were to be relieved only by blood-letting.

After this no local treatment was resorted to until the year 1824, when the tumor having increased to a still larger size, another attempt to restrain its growth by pressure was instituted under the direction of Sir ASTLEY COOPER; but with no more favorable result than formerly.

In the end of June, 1826, the disease having made still further progress, Sir Astley Cooper was again consulted, and by him a ligature was applied (at four different times) round each of the four principal arteries by which the tumor was supplied. The result of these operations was a slight diminution in the size of the tumor, and some relief from pain: but even this favorable change was of short duration. In the course of the winter of 1827, the tumor again grew larger, and the painful sensations returned with redoubled violence, attended with a constant sense of weight over the eyes, and excessive depression of spirits. Occasionally there were paroxysms of pain still more violent than what was usually experienced, and followed by a state of extreme languor and exhaustion.

Miss —— remained precisely in this state, except that the tumor continued slowly to enlarge, until the 9th of October, 1828, when she arrived in London, after an absence of many months, and I saw her in consultation with Dr. ROBERTSON, of Northampton. The tumor was now bigger than a large been proved that there is in these cases any actual increase of the anastomosis which naturally exists between the smaller arteries. The phenomena of the disease are sufficiently explained on the supposition of the arteries being simply dilated and elongated, and thereby rendered tortuous; and the very interesting dissection recorded by Mr. MAYO, in the Medical Gazette, vol. i. p. 261, renders it probable that this is the only change that really takes place in the condition of the blood-vessels. 2d. The term aneurism by anastomosis might with equal propriety be applied to some other blood-vessel tumors, certain nævi for example, which nevertheless differ from the disease in question in many essential circumstances.

double walnut, occupying a spot on the right side of the forehead, immediately below the margin of the hairy scalp. When the fingers were applied to it, they received an impression as if it was composed of a mass of tortuous vessels, and a strong pulsation was perceptible in every part of it. The skin covering the tumor was thin, and on some occasions, as in coughing, when the vessels were unusually distended, it appeared as if on the point of bursting. When the scalp was shaved, large and tortuous arteries were to be seen, even from a considerable distance, passing into the basis of the tumor, in every direction, from each temple, from the orbit of the right eye, and over the crown of the head from the occiput. Pressure being made on the two temporal arteries at the same instant, the pulsation of the tumor was perceptibly, but not greatly, diminished. There was a constant sense of weight and pain in the forehead, and the latter was very much aggravated by pressure on the tumor, especially on a particular spot towards its upper edge.

The sufferings of the patient were such that she was willing to submit to any plan of treatment which might afford her even a chance of being relieved. On considering the subject, it appeared to Dr. Robertson and myself that there was no reason to expect advantage from any further attempt to obliterate the arteries by which the tumor was supplied with blood, nor indeed from any operation which had not for its object the complete extirpation and removal of the diseased structure. But the attempt to accomplish this object by means of the knife, would necessarily be made at the risk of a most alarming hemorrhage; and the application of the actual cautery, or of caustic, would not only be uncertain as to the result, but, if carried to a sufficient extent completely to answer the intended purpose, might occasion such injury to the bone and periosteum as would be productive of much subsequent inconvenience, if not actual danger, to the patient. Under these circumstances Dr. Robertson immediately assented to the proposal which I made, that I should endeavour to extirpate the tumor by means of ligatures, so applied as to produce the complete strangulation of it at its base. There seemed to be at any rate no more effectual nor any safer method of proceeding; but, even with respect to this, it was impossible not to experience, in the first instance, considerable apprehensions as to the loss of blood which might take place on the separation of the slough. These apprehensions were, however, greatly diminished, if not altogether removed, in consequence of the conviction which we felt that the unusual dilatation of the principal arteries of the scalp was to be regarded as the effect, and not the cause, of the morbid growth of the smaller vessels, and as being likely to subside immediately on the tumor being destroyed.

A further consultation having been held with Mr. Keate, and afterwards with Sir Astley Cooper, and both these gentlemen having agreed in opinion with Dr. Robertson and myself, I proceeded to perform the operation, on which we had determined, on Wednesday the 15th of October, in the following manner:

A long steel needle, the length of which was about double the diameter of the tumor, was passed between it and the periosteum, penetrating the skin on each side. By means of this needle the tumor was raised as much as possible, and a second needle was introduced in the same manner, but beneath, and at right angles to, the first. A very strong silk ligature was then bound several times round the base of the tumor, below the needles, as tight as it could be drawn. The tumor immediately assumed a purple colour, as if in a state of strangulation. The operation occasioned great pain, both at the time and afterwards; but, from the instant of the ligature having been applied, the peculiar sufferings occasioned by the disease were at an end.

In the evening, the pulse being strong, the skin hot, and the pain caused by the ligature very severe, some blood was taken from the arm.

October 16th.—The pain was somewhat abated, the tumor had assumed a dark colour, and had begun to shrink.

17th.—The tongue was furred, the pulse hard and frequent, and the skin hot. More blood was taken from the arm.

18th.—All the arteries entering the tumor had either ceased to pulsate or pulsated less strongly than before, with the exception of those at the upper part. Concluding from this last circumstance that the strangulation was not every where complete, and that a still greater degree of compression was necessary, I armed one of the needles with a strong double ligature, then drew it through, and having removed the needle, tied the ligatures one on each side.

20th.—The other needle was armed in the same manner, and by means of it another double ligature was passed through the base of the tumor, and tied like the former one.

22d.—The slough had begun to separate at its edges, and all severe pain had ceased. The pulsation of the arteries at the upper part was greatly diminished.

26th.—The slough came away without the smallest hemorrhage. Dry lint, with stripes of adhesive plaster over it, was applied to the ulcerated surface.

In the course of a few days the ulcer had assumed a healthy appearance, and had begun to granulate.

The appearance of the ulcer was very carefully watched, and two or three times the nitric acid was applied to some spots on its surface, in which there was an appearance that led Mr. Keate and myself to suspect that there might be a disposition to reproduce the original disease. The sloughs made by the nitric acid soon separated; the sore continued to heal, and the pulsation of the arteries in the neighbourhood to diminish.

December 2d.—The cicatrix was completely formed, and nothing unusual was to be observed except that between it and the eyebrow there was a slight appearance of fulness, manifestly depending on the skin at this part having been for a long time much distended, and having not yet returned to its original dimensions. There was no more pulsation in the arteries, which had formerly been so much enlarged, than in those of the other side of the forehead; and the patient was free from pain and all other inconvenience.

---

*Case of Intestinal Obstruction successfully treated by Mechanical Means.* By Alexander Russel Duguid, m.d. Kirkwall. (*Edinburgh Med. and Surg. Journal.*)

On the 10th December, 1828, three o'clock p.m. I was called to visit J. H., a young man, about twenty years of age, who had suffered severely, since the afternoon of the 8th, from obstinate constipation, attended with occasional paroxysms of violent pain in the abdomen, generally ending in efforts to vomit. On the 9th he had taken two ounces of castor oil, and a common domestic enema had been administered; but the greater part of the oil had been vomited, and neither of these expedients produced the smallest discharge from the bowels, or any mitigation of his sufferings. He had also used the warm bath. Abdomen hard, and very much distended; pulse 100.—Emit$^{r}$ Sanguis ad ℥xxiv. et habt. Haust. c. Tinct. Opii gutt. L.

In two hours the opiate began to have its due effect, and he experienced almost total cessation of the abdominal pain and retching. This appearing a favorable opportunity for the exhibition of a purgative, an ounce of castor oil was given every half hour, till four ounces were taken without being rejected; and I also threw up a strong enema of senna and salts. The latter having come away in half an hour without any trace of feculent matter, it was frequently repeated, but always with the same result. At nine P.M. he was again bled to sixteen ounces, and an emulsion containing two drops of croton oil was given every half hour. The greater part of this remained on the stomach, but at twelve P.M. no evacuation had taken place from the bowels, though repeated attempts had been made; and the immunity from suffering which the patient had enjoyed while under the influence of the opiate was now at an end, the paroxysms of pain having returned with aggravated violence.

Being twelve miles from home, and from the nearest supply, I was unavoidably confined to the chance which remained to my patient from mechanical means alone, since no new medicinal expedients could be resorted to. Dilatation of the rectum with warm water, thrown up by Weiss's powerful syringe, was next resorted to. As soon as about a pint and a half were thrown up, he complained of much pain and distention, and it was returned with great force in spite of my efforts to prevent it, and without any trace of feculent matter. This was frequently repeated, with the same result. I then introduced the elastic tube of Weiss's instrument, well oiled, about ten inches into the rectum, and, finding an obstruction to its further passage, I fitted the syringe to its extremity, and continued to exhaust the air for a minute or two; but this having no effect, I attempted to push the tube past the obstruction. After some difficulty, and repeated trials, I gained a few inches, when all at once, to my great satisfaction, the resistance was overcome, and a copious discharge of very fetid flatus, with some liquid feces, took place through the tube, with almost instant relief of the distention and pain of the belly. I then fitted the syringe to the extremity of the tube, and pumped out a large quantity of feculent matter, of the appearance and consistence of yeast. When this was too solid to pass through the tube and syringe, so as to choke the instrument, a quantity of warm water was thrown in, and the pumping process resumed. In this way a great accumulation of feces was brought away, with total relief of all the symptoms. Upon withdrawing the tube, the cause of the obstruction (which indeed I had previously surmised,) became very apparent, by the thin streaks of hardened feces with which the tube was coated on various parts of its surface, and which were confined to that portion of it which had passed the region, about ten inches from the anus, where the great difficulty of introduction had been experienced. An opiate enema was now administered, and I left my patient at eight A.M. of the 11th. A purgative mixture of senna and salts was sent him, which operated well, and left him convalescent.

Obstruction of the intestinal canal is a disease so serious in its nature, of so frequent occurrence, and often so unmanageable by the ordinary treatment of clysters and purgatives, that any available addition to the resources of our art, free from danger, and of easy application, cannot fail to be acceptable to the professional public. So far as I know, the expedient, the good effects of which in one instance I have detailed, has been hitherto untried; nor was I aware that it had ever been proposed, until long after the occurrence of the above case. At all events, it has no place, as applicable to desperate cases, in the systems of medicine which are generally put into the hands of young practitioners. Our principal reliance is placed on purgatives, mechanical dilatation with warm water, and tobacco in the form of smoke or infusion. When excessive vomiting, indicating the inversion of the peristaltic motion, and great swelling of the abdomen, have taken place, many inconveniences attend the use of purgatives; for, even when the difficulty of getting them retained on the stomach is surmounted, and their influence is duly exerted on the bowels, they seem to increase the distention by forcing down the contents of the upper intestines, and thus produce a state which is unfavorable for the action of the abdominal muscles in the expulsion of feces. On this account, the croton oil, the most powerful of them all, seems better adapted to cases where the costiveness is owing rather to torpor than to obstruction of the bowels. The application of the tobacco enema, though sometimes effectual, is also in some degree objectionable, because it is seldom had recourse to unless as a last resource; and when great prostration of strength has already taken place, its debilitating effects are confessedly not free from danger. If, then, a great proportion of cases of ileus be caused by a hardened ball of feces in the colon, at no great distance from the anus, perhaps at the sigmoid flexure, a different, or at least an additional expedient, when all others fail, ought to be employed. A hollow bougie of elastic gum, with its point conical and perforated on the sides, ought to be introduced into the rectum; and the seat of the obstruction having been thus ascertained, by cautiously applying additional force, it may be pushed through the offending substance, if merely hardened feces, and then the management of the case will become comparatively simple. It will be necessary, no doubt, in such an operation, to be cautious. It is possible that the point of the tube may become entangled in the coats of the intestine, and an obstacle to its farther progress may be thus produced; but the resilience of the instrument will sufficiently indicate the nature of the difficulty, and communicate a very different feeling from the resistance afforded by an inelastic substance such as hardened feces. Where the obstruction arises from calculus, no benefit is to be expected from the measure I have detailed; and even the natural contents of the intestines may become so indurated as to form an obstacle almost equally insurmountable, at least by an instrument of so soft and yielding a nature as can be safely employed for such a purpose; but it may be presumed that such cases are comparatively rare, and that in general the cause of the disease may, with care and perseverance, (more especially if within the efficient reach of the tube,) be eventually overcome. Of the bad effects which might by possibility result from such a practice, I should wish to speak guardedly, my experience being much too limited. It is certainly easy to conceive that any violent and repeated efforts might produce injury to the mucous coat of the intestine, perhaps dangerous inflammation. But it is not probable that such accidents will occur in the hands of any experienced person; and that they are not peculiarly liable to happen, may be inferred from the freedom which is used with these parts, in introducing bougies for the dilatation of permanent stricture, in which cases, from the morbid irritability already existing, accidents might more naturally be expected to follow.

Several months ago, a similar case was detailed in the Medical and Physical Journal, occurring at Genoa, in which, after the failure of every other remedy, the disease was happily overcome by exhausting the air in the rectum, beneath the seat of the obstruction; but it may safely be affirmed that in many instances so feeble an expedient would be altogether nugatory. It forms, however, the nearest approximation to the practice above described which has as yet occurred to me, and was unquestionably the hint which led me to the adoption of a bolder and more effectual measure.

---

*Case of Wound of the Femoral Artery, successfully treated.* By William G. Dickinson, m.d. of Franklin, Tennessee. (*American Journal of Medical Sciences.*)

At ten p.m. on the night of the 25th March, 1828, I was called in haste to see Mr. James C. Hill, merchant, a young gentleman of moral habits and fine health, who, it was said, had been stabbed and was dying. I found him, ten minutes after the reception of the injury, lying on his back, covered with blood, and with both hands holding the edges of the wound firmly in contact. The wound was a little below the external abdominal ring, and just exterior to the spermatic cord of the right side, and nearly in the direction of a line drawn from the upper portion of the symphysis pubis, to the inferior spinous process of the ilium. A large tumor extended from the ilium to the pubes, and the right half of the scrotum was distended to four times its natural dimensions, of a dark purple colour.

Drs. R. H. Campbell and T. Stith having come to my assistance, I made an incision, two inches in length from the wound, towards the symphysis pubis and over the most prominent part of the tumor. After going as deep as prudence seemed to sanction, and finding nothing but an injected cellular structure, another incision, three inches in length, was made towards the spinous process of the ilium, in the direction of Poupart's ligament. So soon as the skin and cellular tissue were divided by this incision, the blood gushed forth with considerable velocity. I instantly passed my finger into the opening, and, guided by the warm jet of blood, placed it on the femoral artery. About one third of the artery's circumference was divided directly at the point of its exit, from under Poupart's ligament. This ligament was also divided at its lower margin, apparently to an extent equalling the breadth of the instrument by which the wound was inflicted.

The wound being cleansed, it was evident that the hemorrhage was completely controlled by the pressure of my finger: in fact, it was so entirely suppressed, that Dr. Stith proposed tò confide the case to compresses and a bandage. To secure the vessel conveniently, it was found necessary to make an incision an inch and a half in length, in the direction of the femoral artery. Constant pressure being necessary to prevent a recurrence of hemorrhage, some difficulty was encountered in passing the ligature. It was, however, finally effected by a director and needle, which last instrument was firmly grasped in a pair of small forceps. The ligature being divided, one portion was carried as high as possible, and firmly tied; the other as low as the detachments would permit, and also firmly tied. The ligatures used were of silk, in preference to the animal ligatures suggested by Dr. Stith.

All pressure being removed, the wound neatly sponged and left exposed for some time, and no hemorrhage recurring, the parts were brought in contact, and secured by adhesive strips, over which a compress and bandage were applied. The patient's legs were flexed and well supported, and his situation rendered as comfortable as circumstances would permit.

He was left to repose at three o'clock A.M. after taking Tinct. Opii gutt. xxx.; pulse ninety-five. He had taken during the operation ℥ss. Camph. Tinct. Opii, and some undiluted spirit, which he said was tasteless.

26th.—Slept none since the operation; complains of pain in different parts of the body, but more particularly in the knee and muscles of the leg of the right side; no appreciable difference in the temperature of the two extremities; pulse ninety-two. At intervals during the day, took a little chicken water, and at night a cup of tea. No pulsation in the leg, and in the evening rather cold. Gentle friction ordered. At night took ℥ss. Epsom salts in three doses. At ten o'clock at night, slight pulsation at the internal ankle was perceptible to Dr. O'Bryan, who was with him.

27th.—Slept three hours during the night. Salts operated well; felt relieved by their operation.

Ten o'clock A.M.—Pulse eighty-five, and quite perceptible at the ankle; still complains of pains in the joints and muscles of the right extremity.—Ordered friction and passive motion of the ankle and knee joints. A cup of tea for breakfast, and light soup at noon.

Four o'clock P.M.—Pulse ninety-six. Too much company. Slept half an hour in the evening.—Eight o'clock: Pulse eighty-five. Took a cup of tea. Being restless, Tinct. Opii gutt. xxx. were given at twelve o'clock.

28th.—Slept two and a half hours after taking the opiate. Pulse this morning eighty and regular. Ordered for breakfast tea and a little toasted bread.

Six o'clock P.M.—Pulse seventy-five.

29th.—Passed a restless night. At four o'clock A.M. took Tinct. Opii gutt. xxx.; after which he slept two hours.—Eight o'clock A.M., pulse 100.—Ten o'clock, ordered salts, which operated well, and afforded considerable relief.

Six o'clock P.M.—Pulse eighty. Eight o'clock, wound examined: it had united throughout, except at the exit of the ligatures and the angle of junction of the incisions, at which point there was a suppurating surface, about an inch and a half long, and half an inch broad, presenting pus of a healthy character. The edges of the wound were drawn in contact by adhesive slips. Ordered Tinct. Opii gutt. xl. to be taken at eleven o'clock.

30th.—Slept but little, notwithstanding the opiate.—Six o'clock, pulse 100. Ordered coffee for breakfast, and a little rice at noon. Slept several hours during the day.—Four o'clock P.M, pulse eighty-four, and soft.

31st.—At ten o'clock last night took Tinct. Opii gutt. xxx., but passed a restless night. At six o'clock this morning had a natural evacuation from his bowels.—Eight o'clock, disposed to sleep; pulse 108, and vibrating. Diet as yesterday. Wound looks well.

April 1st.—Rested better last night than he has done since the accident; took no opiate.—Six o'clock A.M., pulse eighty, and soft. Took Epsom salts ℥ss. which operated well. Diet as yesterday. The wound looks well, but the pus a little tinged with blood.

2d.—At one o'clock A.M. took Tinct. Opii gutt. xxv., in part to relieve a disposition to cough, which has become troublesome, and irritates the wound. The pus considerably tinged with blood, but the wound healthy in appearance; pulse 100, with some heat of the skin. Diet as usual.

From this period his improvement was gradual, until the 11th, when the lower ligature came away: the other ligature remained until the 18th, when it was withdrawn without difficulty. The wound presented a healthy aspect until the 27th, at which time its appearance was irritable, and the discharge unhealthy. This change was produced by circumstances of an exciting and disagreeable nature. Ordered a more nourishing diet.

From this to the 31st the improvement was rapid, at which time the wound was entirely cicatrised.

Mr. Hill has since enjoyed uninterrupted health, and experiences no inconvenience from the accident; and is actively engaged in his usual avocations.

---

### MISCELLANEOUS.

*Mode of preserving Specimens of Morbid Anatomy.* By John S. Gaskoin.—Mr. Gaskoin recommends the following means for preserving the appearances of diseased parts:

"Having removed the diseased part from the body, it should be as little handled or dissected as possible, especially when the effects of inflammation, congestion, &c. are to be preserved, as the blood may be pressed from, or disturbed in, the minute vessels. Let the blood which may have escaped from cut vessels, be gently washed off from the surface by a solution of the muriate of ammonia, or be absorbed by a soft sponge, lightly applied. The part should then be wrapped with care in old linen, and be so immersed in one part of a saturated solution of the muriate of ammonia, (sal ammonia of commerce,) and two of rectified spirit of wine. After two or three days the linen may be removed, and the part restored to the fluid.

"Should the preparation be large, or, from the nature of the disease, contain a large quantity of aqueous fluid, then an additional portion of the muriate of ammonia in powder should be added, to meet the excess of aqueous menstruum.

"The time necessary for maceration will mainly depend upon the size of the part to be preserved; but, generally, from ten to fifteen days will be found to be sufficient, although nothing can be lost by an extension of that time. Being taken from the macerating fluid, it should be again washed in a solution of the muriate of ammonia, then dissected as much as requisite, and be 'put up' at once, in equal quantities of a saturated solution of the above salt in distilled water and rectified spirit of wine. I should observe that, in these proportions, the part is somewhat corrugated, which is not the case if *one third* of the saline solution be used with two of the spirit; yet, in the former quantities, I have some reason to think the appearances of disease may be more securely preserved."

This solution, Mr. G. says, seems to have the property of fixing the blood in the extreme ramifications, without constringing the vessels themselves; while rectified spirit, corrugating the delicate membranes of the minutest vessels, repels their contents into the larger, the thicker coats of which are easily acted on, and thus reduces the appearances of inflammation, &c.

